# Risk assessment in a Chinese cohort of 96 318 females undergoing opportunistic cervical cancer screening

**DOI:** 10.1093/oncolo/oyaf197

**Published:** 2025-07-14

**Authors:** Jing Zhao, Wenhan Li, Zehua Wang, Weichao Liu, Si Sun, Liying Wu, Shi Du, Guoqing Li, Zhongya Pan, Dingyu Chen, Pinglan Yang, Wuliang Wang, Liqiong Cai, Bangxing Huang, Jing Cai

**Affiliations:** Department of Obstetrics and Gynecology, Union Hospital, Tongji Medical College, Huazhong University of Science and Technology, Wuhan 430022, China; Department of Obstetrics and Gynecology, Union Hospital, Tongji Medical College, Huazhong University of Science and Technology, Wuhan 430022, China; Department of Obstetrics and Gynecology, Union Hospital, Tongji Medical College, Huazhong University of Science and Technology, Wuhan 430022, China; Department of Gynecology, Maternal and Child Health Hospital of Hubei Province, Wuhan 430022, China; Department of Obstetrics and Gynecology, Union Hospital, Tongji Medical College, Huazhong University of Science and Technology, Wuhan 430022, China; Department of Obstetrics and Gynecology, Union Hospital, Tongji Medical College, Huazhong University of Science and Technology, Wuhan 430022, China; Department of Obstetrics and Gynecology, Union Hospital, Tongji Medical College, Huazhong University of Science and Technology, Wuhan 430022, China; Department of Obstetrics and Gynecology, Union Hospital, Tongji Medical College, Huazhong University of Science and Technology, Wuhan 430022, China; Department of Obstetrics and Gynecology, Union Hospital, Tongji Medical College, Huazhong University of Science and Technology, Wuhan 430022, China; Department of Obstetrics and Gynecology, Union Hospital, Tongji Medical College, Huazhong University of Science and Technology, Wuhan 430022, China; Department of Obstetrics and Gynecology, Union Hospital, Tongji Medical College, Huazhong University of Science and Technology, Wuhan 430022, China; Department of Gynecology, The Second Affiliated Hospital of Zhengzhou University, Henan Provincial Clinical Research Center for Gynecologic Oncology, Zhengzhou 450014, China; Department of Obstetrics and Gynecology, Union Hospital, Tongji Medical College, Huazhong University of Science and Technology, Wuhan 430022, China; Department of Pathology, Union Hospital, Tongji Medical College, Huazhong University of Science and Technology, Wuhan 430022, China; Department of Obstetrics and Gynecology, Union Hospital, Tongji Medical College, Huazhong University of Science and Technology, Wuhan 430022, China

**Keywords:** cervical cancer screening, risk estimation, validation, HPV genotypes, risk-based management

## Abstract

**Objective:**

To assess CIN3+ risk in a Chinese cohort of outpatients undergoing contesting screening and to evaluate the portability of the American Society for Colposcopy and Cervical Pathology (ASCCP) risk-based management, which was primarily developed using data from the Kaiser Permanente of Northern California (KPNC) cohort.

**Methods:**

Females aged 25-65 years who were screened with cytology and high-risk human papillomavirus (hrHPV) co-testing between 2011 and 2020 at Wuhan Union Hospital (WHUH) were retrospectively studied. The risks of immediate and 3-year CIN3+ were estimated via prevalence-incidence mixture models. Portability was evaluated via the ratio of the observation risk in the WHUH cohort to the expected risk in the KPNC cohort (O/E) and its 95% CI.

**Results:**

A total of 96 318 females were included, and 16.83% of the women tested hrHPV-positive at initial screening, who had a CIN3+ immediate risk of 14.14%. The CIN3+ immediate risk varied between subgroups of positive HPV16 (34.09%), HPV18 (13.38%), other HPV types (6.71%), and negative hrHPV (0.12%). Compared to the KPNC cohort, our cohort exhibited a significantly higher CIN3+ immediate risk (1.42% vs 0.46%; O/E, 3.09; 95% CI, 2.92-3.26) and disproportionately increased cancer immediate risks in most subgroups requiring immediate colposcopy or treatment, as well as higher 3-year CIN3+ risks in women with hrHPV-negative ASC-US/NILM. Yet, the action threshold suggested by ASCCP, a CIN3+ immediate risk of 4%, showed good portability to our cohort.

**Conclusions:**

Despite the higher risks in our cohort, the ASCCP clinical action threshold remains portable. For women with minimal abnormalities or normal results, shortened follow-up intervals should be considered.

**Implications for Practice**

CIN3+ risks in opportunistic screening populations remain understudied, and the risk-based management recommendations have yet to be well validated outside of the United States, particularly in China. This study is the first to report CIN3+ risks in a Chinese cohort using prevalence-incidence mixture models. It supports the portability of ASCCP’s 4 % CIN3 + risk threshold and suggests shorter follow-up strategies for women with ASC-US/NILM.

## Introduction

Globally, there were 661 021 estimated new cervical cancer cases in 2022, with an age-standardized incidence rate of 14.1 per 100 000.^1^ In China, it was estimated that approximately 150 700 new cases of cervical cancer were diagnosed and that 55 700 women died of this disease in 2022.^2^ China has included screening for this disease in the national public health services program since 2009, and the coverage of cervical cancer screening has been largely improved across the nation, albeit rather inadequate in rural regions (33.2%-63.7%).^3,4^ HPV vaccination and cervical cancer screening are recommended by the World Health Organization for eliminating this HPV-caused malignancy (incidence rate less than 4 per 100 000). The current mainstream screening methods involve HPV testing and cytology on a global scale, often in combination either concurrently (co-testing) or sequentially (upfront HPV followed by cytology triage). Based on the results of initial screening, follow-up at certain intervals, referral to colposcopy, or treatment are recommended to detect and manage precancerous lesions in a timely manner, thereby preventing participants from developing invasive cancers.

In the 2019 American Society for Colposcopy and Cervical Pathology (ASCCP) consensus guidelines, the management principle of abnormal cervical cancer screening results has evolved from being result-based to being risk-based, ie, equal management for equal risk, wherein the risk mainly refers to the immediate risk of cervical intraepithelial neoplasia grade 3 or higher (CIN3+) that is established at the current visit and the CIN3+ cumulative risk established at 3 or 5 years after the current visit.^5^ In specific circumstances, cancer risk is also considered. For example, for women with HPV-negative/ASC-H and HPV-negative/AGC, we need to refer to cancer risk because they have a low CIN3+ risk but an elevated cancer risk. According to these guidelines, an immediate CIN3+ risk of 4% is the threshold for clinical action (4%-24%, colposcopy; 25%-59% treatment, or colposcopy, 60%-100%, treatment), and the cumulative CIN3+ risk is used to determine the return interval in women who don’t hit the clinical action threshold. The management consensus guidelines are supported by risk estimates in a cohort of 1.5 million females aged 25-65 years who were screened in the Kaiser Permanente of Northern California (KPNC) with co-testing, and the action thresholds are determined based on the risks in reference populations, who should take action according to the 2012 ASCCP guidelines. For example, the colposcopy referral threshold is rooted in the immediate risk of CIN3+ in women with HPV-positive/ASC-US (4.4%).^5^

Theoretically, compared with result-based management, risk-based management allows for a more precise triage of women with positive results in HPV testing and/or cytology examinations. However, the integration of risk-based management into clinical screening programs has at least 2 prerequisites, ie, accurate risk estimates in local populations and reasonable action thresholds matching local resources. The ASCCP launched guidelines for risk-based management in 2019 until the time that screening data from a large cohort with long follow-up periods were available. The KPNC risks and risk-based management have been validated in 3 US cohorts, including the Centers for Disease Control and Prevention’s National Breast and Cervical Cancer Early Detection Program (CDC PNBCCEDP), the Onclarity Human Papillomavirus Trial, and the Addressing the Need for Advanced HPV Diagnostic study and in the New Mexico HPV Pap Registry and revealed satisfying portability.^6,7^ However, the generalization regarding this situation is complex, as the immediate and cumulative CIN3+ risk varies based on a wide range of factors, from screening participants to screening methods to management of screening results. In countries and areas with higher cervical cancer prevalences, screening is more opportunistic than organized, and opportunistic screening populations generally demonstrate higher (pre)cancer risks than average populations.^8,9^ Herein, we estimated the immediate and 3-year CIN3+ risks, along with CIN2+ and cancer risks, in an opportunistic screening cohort of approximately ten thousand women undergoing co-testing at Wuhan Union Hospital (WHUH), China, by using the prevalence-incidence mixture models, which were identical to the ASCCP guidelines.^10^ Based on the estimates, projected outcomes regarding efficiency, benefits and harm from different colposcopy and treatment referral thresholds were assessed, and the portability of KPNC risks and risk-based management to our cohort was evaluated. These efforts have strived to offer references for applying risk-based management to opportunistic screening populations.

## Methods

### Study population and data collection

We retrospectively collected all of the data of women who were screened with co-testing from August 1, 2011 (the very beginning of introducing contesting at WHUH) to December 31, 2020, including the age at the first screening visit, the results of every round of cytology and HPV tests, the histological diagnosis that was established when the women were biopsied, and the history of screening and gynecological surgery of each woman, especially with respect to hysterectomy. For the final analysis, individuals aged 25-65 years without a history of CIN2+ or hysterectomy before the first visit based on medical records were included, and those who with missing cytology results or unsatisfactory cytology were excluded. In the study population, women who had been vaccinated against HPV were rare because the local vaccination program was not launched at that point in time. The study protocol was approved by the Ethics Committee of Tongji Medical College, Huazhong University of Science and Technology (No: 2021-S353).

### Cytology and HPV co-testing

Liquid-based cytology examination (SurePath, BD) and HPV genotyping (Shanghai Tellgen Life Science Co., Ltd.) were conducted in parallel. The screening methods were consistent throughout the study period. The cytology slides were manually viewed by experienced cytopathologists and interpreted according to the 2001 Bethesda System classifications: negative for intraepithelial lesion or malignancy (NILM); atypical squamous cells of undetermined significance (ASC-US); atypical squamous cells, cannot exclude high-grade squamous intraepithelial lesion (ASC-H), low-grade squamous intraepithelial neoplasia (LSIL), high-grade squamous intraepithelial neoplasia (HSIL), squamous cell carcinoma (SCC), atypical glandular cells of uncertain significance (AGC), adenocarcinoma in situ (AIS), and adenocarcinoma (AC). In the present study, the HSIL+ lesions included HSIL, AIS, SCC, and AC.

The HPV testing was PCR-based and ran on a Luminex 200 (Merck Millipore) platform for detecting 17 hrHPV types (HPV16, 18, 26, 31, 33, 35, 39, 45, 51, 52, 53, 56, 58, 59, 66, 68, and 82) separately.^11^ This method was approved by the China Food and Drug Administration for cervical cancer screening and has demonstrated comparable effectiveness to that of the Cobas HPV test in terms of detecting CIN2+ in women with positive HPV results, with a sensitivity of 94.59% (95% CI, 86.91%-97.88%) vs 93.24% (95% CI, 85.14-97.08) and a specificity of 72.79% (95% CI, 64.77-79.57) vs 73.53% (95% CI, 65.54%-80.22%).^12^

### Clinical management

Women with abnormal screening results were managed according to internal center guidelines, which were mainly in line with the 2006 and 2012 ASCCP guidelines.^13,14^ Women who met one of the following criteria were subjected to colposcopy examination and biopsy: (1) HPV16 or HPV18 positive, any cytology; (2) other HPV-positive, ASC-US+ cytology; or (3) HPV-negative, LSIL+ cytology. For women with other HPV-positive and NILM, a 1-year return was recommended. When considering the relatively high incidence observed in our population and the opportunistic screening setting with poor participant adherence, women with negative screening results (HPV-negative and NILM) were also recommended to return within 1 year.

In colposcopy examinations, we inspected the cervix with the aid of the white acetic acid test and the Schiller test and performed cervical biopsies on suspicious regions or at positions of 3:00, 6:00, 9:00, and 12:00 at the squamocolumnar junction of the cervix. In cases of poor cervix exposure or the presence of abnormalities involving glandular cells revealed via cytology, additional endocervical curettage would be performed. The samples were then pathologically examined to detect cervix lesions, including cervical intraepithelial neoplasia (CIN1-3), SCC, AIS, and AC. The females who were not undergoing colposcopy were counted as not having CIN2+ lesions.

### Statistical analysis

We estimated the risks of cervical precancerous lesions and cancer via prevalence-incidence mixture models, just as the same used in the KPNC study. WHUH didn’t use the ASCCP web app directly for risk assignments. These models are superior for analyzing cervical cancer screening data over standard statistical methods for risk estimates, as the data are often featured with left censoring (unknown history before the current test) and interval censoring (unknown time of incident cervical lesion occurs between 2 visits).^15,16^ By applying the “PIMixture” R package, we calculated the immediate, 3-year, and 5-year risks of CIN2+, CIN3+, and cancer stratified by cytology and HPV test results via R Studio software, version 3.5.2. To inform clinical action thresholds for current treatment and colposcopy/biopsy, the frequency and immediate risk of each co-testing in the initial screening were calculated to project the following measures of benefits, harm, and efficiency per 100 000 screened patients: the number of patients referred to colposcopy/treatment, the number of patients with CIN3+ who were timely detected/treated (number of test-positive cases for the referral criterion), the number of females with <CIN2 undergoing unnecessary procedures (number of false-positive cases), the number of patients with CIN3+/cancers for whom detection/treatment would be delayed (number of false-negative cases), and the number of colposcopies/treatments needed to detect/treat one CIN3+ (efficiency). Moreover, the ratio of the observed risk in our cohort to the expected risk in the KPNC cohort (O/E) and the 95% CI were calculated to assess the portability of the risk estimates and management.

## Results

### Cohort demographic characteristics and test results

**Figure 1.**
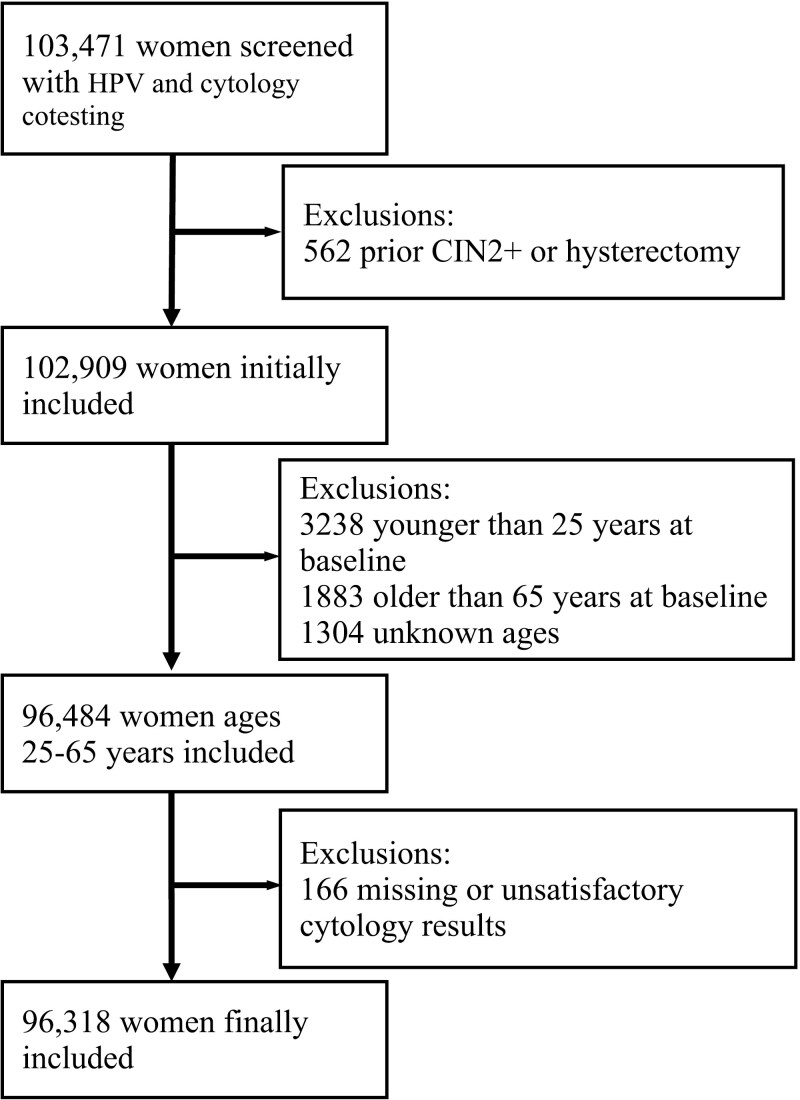
A diagram of the inclusions and exclusions. CIN2+, cervical intraepithelial neoplasia grade 2 or more severe diagnoses.

Between August 2011 and December 2020, 103 471 women underwent cervical cancer screening at our center. Among them, 96,318 women aged 25-65 years (median: 42 years) who underwent cytology and HPV genotyping co-testing met the including criteria and were included in the subsequent risk estimates (Figure 1). Overall, the cohort was underscreened, as 74 193 women (77.03%) had only one single visit during the time period, and only 8861 women (9.20%) were followed up for more than 3 years (Table 1). At the initial screening, 16 215 (16.83%) women tested hrHPV-positive (HPV16, 18.12%; else other HPV18, 5.20%; and other hrHPV, 76.68%), and 20 735 (21.53%) had abnormal cytology (ASC-US, 62.98%; LSIL, 17.34%; AGC, 8.16%; ASC-H, 5.25%; and HSIL+, 6.27%). The hrHPV infection rate in individual age groups varied between 15.6% and 21.4%, with the highest rate observed in the 60-65 years age group (Figure S1). During the study period, 6649 women received colposcopy referrals, and CIN3+ lesions were pathologically diagnosed in 1321 (1.37%) women, including 486 invasive cancers. Among the CIN3+ lesions and cancers that were detected, hrHPV-negative cases accounted for 8.18% and 7.20% of cases, respectively.

**Table 1.** Clinical scenario and round of visits of the WHUH cohort.

Clinical scenario and round of visits	Age^a^ (median (IQR))	*n*	Follow-up^b^ (median [IQR])	*n* with > 3-year follow-up	*n* with > 5-year follow-up
Precolposcopy					
Initial screen	42 (34-48)	96,318	0 (0-0)	8861	3445
Second visit	40 (33-46)	22,125	2 (1-4)	8861	3445
Third visit	40 (33-45)	8862	3 (2-5)	5782	2491
Fourth visit	39 (33-45)	4073	4 (3-5)	3390	1700
Postcolposcopy for <CIN2					
At colposcopy	44 (36-50)	6649	0 (0-1)	808	325
First visit	42 (35-48)	2187	2 (1-3)	808	325
Second visit	41 (35-46)	1017	3 (2-4)	612	243
Third visit	41 (34-46)	527	4 (3-5)	401	183
Posttreatment for CIN2+					
First visit	44 (37-50)	1985	0 (0-1)	270	100
Second visit	42 (35-48)	801	1 (0-3)	270	100
Third visit	41 (35-46)	416	3 (1-4)	211	79

^a^Age are at the time of the initial screening, range 25-65 years.

^b^Follow-up time is defined as time (in years) from the initial screening to the last visit.

*n* indicates numbers of females screened; IQR, interquartile range; CIN, cervical intraepithelial neoplasia.

### Risk estimates

Using the prevalence-incidence mixture models, the immediate, 3-year, and 5-year risks of CIN2+, CIN3+, and cancer were estimated (Table 2). Given the pivotal role of CIN3+ risks in risk-based management and the relatively insufficient follow-up, we mainly focused on CIN3+ immediate risks. Overall, the CIN3+ immediate risk in our cohort was 1.42%, and it varied significantly between women with different screening results. The immediate CIN3+ risk and cancer risk were 14.14% and 5.36%, respectively, in the hrHPV-positive women, compared with 0.12% and 0.04%, respectively, in the hrHPV-negative women. In the subgroup analyses in which the participants were further stratified by their cytology results, the highest risks were observed in those women who tested hrHPV-positive and HSIL+, with an immediate CIN3+ risk of 70.19% and an immediate cancer risk of 30.01%; additionally, even in the hrHPV-negative women, HSIL+ was associated with an immediate CIN3+ risk of 47.66%. In the hrHPV-positive women, the CIN3+ risk increased with increasing severity of cytology abnormalities, ranging from 1.25% to 70.19%. Among them, the hrHPV-positive/ASC-US subgroup, which is the reference used for informing the action threshold of colposcopy/treatment referral by the ASCCP, had an immediate CIN3+ risk of 4.28%. All the immediate CIN3+ risks were lower than 4% in the hrHPV-negative women with any cytology results, except for HSIL+ (47.66%), which was a rarity with only 51 cases (0.06%) in 80 103 hrHPV-negative women.

**Table 2.** Immediate, 3-year, and 5-year risks of cervical lesions in WHUH screening females with unknown screening history.

hrHPV	Cytology	All		CIN2+ risk, %				CIN3+ risk, %				Cancer risk, %			
		*n*	%	*n*	Immed.	3-year	5-year	*n*	Immed.	3-year	5-year	*n*	Immed.	3-year	5-year
Positive	Any	16 215	16.83	1996	24.09	26.12	26.76	1175	14.14	14.88	15.15	439	5.36	5.47	5.53
	HSIL+	1163	1.21	891	88.72	89.19	89.30	705	70.19	70.45	70.48	301	30.01	30.01	30.01
	AGC	416	0.43	99	33.06	35.89	36.85	69	23.31	23.31	23.31	51	17.23	17.23	17.23
	ASC-H	427	0.44	163	45.96	52.52	54.59	94	26.55	29.79	31.41	30	8.55	9.26	9.99
	LSIL	2252	2.34	451	25.35	27.97	28.50	169	9.31	10.57	10.76	25	1.44	1.44	1.44
	ASC-US	2975	3.09	237	12.05	15.10	15.80	83	4.28	4.95	5.18	21	1.02	1.36	1.48
	NILM	8982	9.33	155	3.74	6.61	7.48	55	1.25	2.28	2.76	11	0.26	0.45	0.55
Negative	Any	80 103	83.17	274	0.23	1.03	1.34	146	0.12	0.48	0.61	47	0.04	0.15	0.19
	HSIL+	137	0.14	72	67.92	67.92	67.92	51	47.66	47.66	47.66	18	16.82	16.82	16.82
	AGC	1277	1.33	15	1.61	3.59	4.05	13	1.49	2.81	3.14	9	1.12	1.74	1.94
	ASC-H	661	0.69	27	4.90	6.31	6.94	14	2.55	3.04	3.60	4	0.79	0.79	0.79
	LSIL	1343	1.39	47	4.25	5.55	5.86	19	1.71	2.16	2.27	2	0.19	0.19	0.19
	ASC-US	10 084	10.47	39	0.81	1.68	2.08	16	0.02	0.66	0.94	7	<0.001	0.32	0.45
	NILM	66 601	69.15	74	<0.001	0.66	0.96	33	<0.001	0.28	0.39	7	<0.001	0.06	0.09

To explore the value of HPV genotyping in risk estimates, the immediate CIN3+ and cancer risks were analyzed in the women infected with HPV16, HPV18, or other hrHPV (Table 3). In these 3 subgroups, the immediate CIN3+ risk was 34.09%, 13.38%, and 6.71%, respectively, and the immediate cancer risk was 14.32%, 10.13%, and 1.15%, respectively. In the women with HPV16 or HPV18 infection, significant cancer risks were also observed in those with NILM cytology (0.61% and 0.72%, respectively), which were even higher than that in the other hrHPV/LSIL groups (0.53%), thereby supporting the referral of all HPV16/18 positive women to colposcopy or expedited treatment (if risks also exceed the treatment thresholds). In addition, we observed an immediate CIN3+ risk of 2.21% in the other hrHPV/ASC-US women, which did not hit the risk threshold of 4% for colposcopy referral recommended by the ASCCP 2019.

**Table 3.** CIN3+ and cancer immediate risks in females with detected HPV16, HPV18, or other hrHPV.

hrHPV detected	Cytology	*n*	%	CIN3+ cases	CIN3+ immed. risk, %	Cancer cases	Cancer immed. risk, %
HPV16	Any	2938	18.1	718	34.09	299	14.32
	HSIL+	617	3.8	442	82.46	217	40.49
	AGC	88	0.5	31	45.59	22	32.35
	ASC-H	183	1.1	65	31.14	25	15.59
	LSIL	413	2.5	100	28.08	15	4.35
	ASC-US	449	2.8	48	14.67	16	4.47
	NILM	1188	7.3	32	3.43	4	0.61
HPV18	Any	843	5.2	74	13.38	56	10.13
	HSIL+	62	0.4	42	77.78	34	62.96
	AGC	135	0.8	19	18.63	14	13.73
	ASC-H	18	0.1	4	28.57	2	14.29
	LSIL	96	0.6	5	6.76	3	4.05
	ASC-US	129	0.8	1	1.11	1	1.11
	NILM	403	2.5	3	0.78	2	0.72
Other	Any	12434	76.7	383	6.71	84	1.51
	HSIL+	484	3.0	221	53.27	50	12.11
	AGC	193	1.2	19	15.08	15	11.90
	ASC-H	226	1.4	25	13.45	3	1.75
	LSIL	1743	10.8	64	4.52	7	0.53
	ASC-US	2397	14.8	34	2.21	4	0.28
	NILM	7391	45.6	20	0.53	5	0.14

### Projected outcomes from different colposcopy/treatment referral thresholds

We subsequently evaluated the projected benefits (number of CIN3+ patients referred), harm (number of <CIN2 patients referred and number of CIN3+ or cancer patients with delayed referral), and efficiency (number of patients referred per one CIN3+ detected) with different colposcopy and treatment clinical action thresholds. The results are shown in Table 4, with the screening results ranked according to the estimated immediate CIN3+ risks that serve as assumed reference thresholds. As the action threshold decreased from 70.19% (hrHPV-positive/HSIL+ as a reference) to 0.02% (hrHPV-negative/ASC-US as a reference), the number of referrals and the number of detected CIN3+ individuals increased, and the number of CIN3+ and cancer patients with delayed referrals decreased, with the number of patients referred per CIN3+ increasing from 1.42 to 18.57.

**Table 4.** Projected outcomes from different colposcopy/biopsy referral thresholds per one hundred thousand screening participants.

hrHPV result	Cytology result	%^a^	Immed. CIN3+ risk,^b^ %	Immed. CIN2+ risk,^c^ %	Immed. cancer risk,^d^ %	*n* of patients referred^e^	*n* of CIN3+ patients referred^f^	*n* of <CIN2 patients referred^g^	*n* of CIN3+ patients with delayed referral^h^	*n* of cancer patients with delayed referral^i^	*n* of patients referred per 1 CIN3+^j^
Positive^1^	HSIL+	1.21	70.19	88.72	30.01	1207	848	136	814	248	1.42
Negative^2^	HSIL+	0.14	47.66	67.92	16.82	1350	915	182	747	224	1.47
Positive^3^	ASC-H	0.44	26.55	45.96	8.55	1787	1031	418	631	186	1.73
Positive^4^	AGC	0.43	23.31	33.06	17.23	2217	1132	706	530	112	1.96
Positive^5^	LSIL	2.34	9.31	25.35	1.44	4548	1349	2446	313	79	3.37
Positive^6^	ASC-US	3.09	4.28	12.05	1.02	7650	1482	5174	180	47	5.16
Negative^7^	ASC-H	0.69	2.55	4.90	0.79	8336	1499	5826	163	42	5.56
Negative^8^	LSIL	1.39	1.71	4.25	0.19	9729	1523	7161	139	39	6.39
Negative^9^	AGC	1.33	1.49	1.61	1.12	11 055	1543	8465	119	24	7.17
Positive^10^	NILM	9.33	1.25	3.74	0.26	20 382	1659	17 444	3	0	12.28
Negative^11^	ASC-US	10.47	0.02	0.81	<0.001	30 852	1662	27 828	0	0	18.57
Negative^12^	NILM	69.15	<0.001	<0.001	<0.001	100 000	1662	96 974	0	0	60.18

The HPV and cytology results are arranged in descending order of immediate CIN3 + risks (rows 1-12).

Assume the colposcopy/biopsy threshold is set so that all test results in rows *1* to *r* are all referred to colposcopy/biopsy (or treatment).

Outcome variables *a–j* are defined as followed:

^a^Percentage of females with the test result given in row *r*.

^b^Immediate CIN3 + risk,

^c^immediate CIN2+ risk, and

^d^immediate cancer risk estimated for the test result given in row *r*.

^e^Number of patients referred to colposcopy/biopsy (number of test positive for the referral criterion) per one hundred thousand patients screened = 100 000$\overset{r}{\underset{l=1}{\sum }}\frac{a_{l}}{100\;\%\;}$.

^f^Number of CIN3+ patients referred to colposcopy/biopsy (number of true positives) per one hundred thousand patients screened=$10000\overset{r}{\underset{l=1}{\sum }}\frac{a_{l}}{100\;\%\;}\frac{b_{l}}{100\;\%\;}$.

^g^Number of <CIN2 patients referred to colposcopy/biopsy (number of false positive) per one hundred thousand patients screened = 100 000$\overset{r}{\underset{l=1}{\sum }}\frac{a_{l}}{100\;\%\;}(1\text{-}\frac{c_{l}}{100\;\%\;}).$

^h^Number of CIN3 + patients who will have a delayed referral to colposcopy/biopsy (number of false negatives) per one hundred thousand patients screened = 100 000$\overset{12}{\underset{l=r+1}{\sum }}\frac{a_{l}}{100\;\%\;}\frac{b_{l}}{100\;\%\;}$.

^i^Number of cancer patients who will have a delayed referral to colposcopy/biopsy per one hundred thousand patients screened = 100 000$\overset{12}{\underset{l=r+1}{\sum }}\frac{a_{l}}{100\;\%\;}\frac{d_{l}}{100\;\%\;}$. ^*j*^Number of patients referred to colposcopy/biopsy to detect one CIN3+ (efficiency) =$\frac{e_{l}}{f_{l}}$.

**Figure 2.**
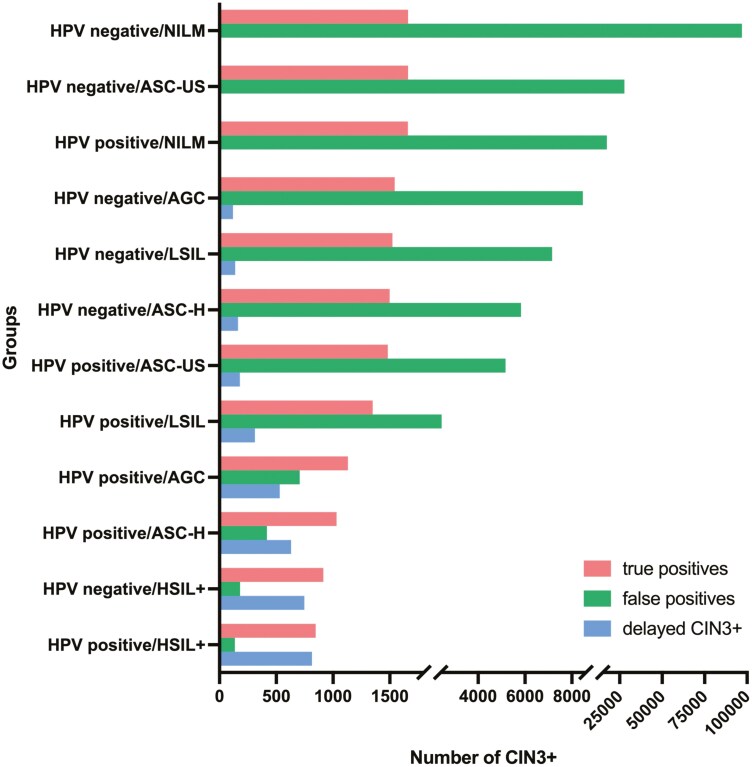
A bar graph showing the number of true positives (CIN3+ patients referred to colposcopy/biopsy), false positives (<CIN2 patients referred to colposcopy/biopsy), and CIN3+ cases with delayed referral per one hundred thousand screening participants when different triage groups are used as colposcopy/biopsy referral thresholds.

For example, when the immediate CIN3+ risk in hrHPV-positive/ASC-US women (4.28%) or the clinical action threshold of the ASCCP 2019 guidelines (4%) was used as the colposcopy/biopsy risk threshold, 7650 women would need colposcopy per 100 000 screened patients screened, of whom 1482 women would be detected with CIN3+ diseases. Meanwhile, 180 (0.18%) women and 47 (0.05%) women would be delayed in the detection of CIN3+ and cancer, respectively, In this case, approximately 5 patients would be referred for colposcopy per one CIN3+ detected, indicating relatively precise candidate selection for colposcopy referrals. When the action threshold is lowered to 1.25% (equivalent to the immediate CIN3+ risk in hrHPV-positive/NILM women), 12 colposcopies would be required to detect one CIN3+ case, resulting in increased unnecessary procedures, while only 3 CIN3+ cases would experience delayed diagnosis. Conversely, raising the threshold to 9% (matching the immediate CIN3+ risk in hrHPV-positive/LSIL women) reduces the colposcopy-to-detection ratio to 3:1 per CIN3+ case, but this approach would delay the diagnosis of 313 CIN3+ cases (Table 4; Figure 2).

### Portability of KPNC risks and risk-based management to the WHUH cohort

The baseline characteristics of the 2 cohorts regarding the main characteristics of the participants, screening methods, and screening results are shown in Table S1. The portability of the CIN3+ and cancer risks and risk-based management that were derived from the KPNC cohort to the WHUH cohort was then analyzed (Table 5). The overall immediate CIN3+ risk in the WHUH cohort was 1.42%, which was significantly greater than that in the KPNC cohort (0.46%; O/E, 3.09; 95% CI, 2.92-3.26). In the 12 subgroups with varied screening results, compared with those in the KPNC cohort, the immediate CIN3+ risk was significantly greater in the women with hrHPV-positive/HSIL+, hrHPV-positive/LSIL, hrHPV-negative/HSIL+, and hrHPV-negative/LSIL in the WHUH cohort, with O/E (95% CI) values of 1.44 (1.38-1.49), 2.18 (1.88-2.53), 1.89 (1.54-2.28), and 1.63 (1.02-2.60), respectively, whereas it was lower in those women with hrHPV-positive/NILM (O/E, 0.59; 95% CI, 0.41-0.84). Remarkably, much poorer portability was observed with respect to immediate cancer risks. Specifically, the immediate cancer risk was significantly increased in 7 subgroups with disproportionately increasing magnitudes compared with CIN3+ risk, and the highest O/E value reached 17.99 (95% CI, 12.19-26.54) in the WHUH women with hrHPV-positive/LSIL.

**Table 5.** Portability of KPNC risks and risk-based management to WHUH cohort.

hrHPV status	Cytology results	KPNC				WHUH								
		Immed. CIN3+ risk, %	Immed. cancer risk, %	3-year CIN3+ risk, %	Recommended management	Immed. CIN3+ risk, %	O/E	95% CI	Immed. cancer risk, %	O/E	95% CI	3-year CIN3+ risk, %	O/E	95% CI
Positive	HSIL+	48.86	4.54	52.06	Treatment/colp.^a^	70.19	1.44	1.38-1.49	30.01	6.61	6.01-7.26	70.45	1.35	1.30-1.41
Positive	AGC	26.27	4.45	32.45	Treatment/colp.^a^	23.31	0.89	0.72-1.09	17.23	3.87	3.01-4.96	23.31	0.72	0.58-0.88
Positive	ASC-H	25.73	0.92	31.41	Treatment/colp.^a^	26.55	1.03	0.86-1.23	8.55	9.25	6.52-13.07	29.79	0.95	0.79-1.14
Negative	HSIL+	25.21	4.32	26.41	Treatment/colp.^a^	47.66	1.89	1.54-2.28	16.82	3.89	2.53-5.87	47.66	1.80	1.47-2.18
Positive	ASC-US	4.45	0.16	6.53	Colp.^b^	4.28	0.96	0.78-1.20	1.02	6.48	4.12-10.18	4.95	0.76	0.61-0.95
Positive	LSIL	4.27	0.08	6.25	Colp.^b^	9.31	2.18	1.88-2.53	1.44	17.99	12.19-26.54	10.57	1.69	1.45-1.97
Negative	ASC-H	3.42	0.69	3.62	Colp.^b^	2.55	0.75	0.43-1.27	0.79	1.13	0.43-2.99	3.04	0.84	0.43-1.62
Negative	AGC	1.07	0.38	1.28	Colp.^b^	1.49	1.40	0.75-2.59	1.12	2.96	1.46-5.97	2.81	2.19	1.10-4.31
Positive	NILM	2.13	0.15	4.11	1-year return^c^	1.25	0.59	0.41-0.84	0.26	1.75	0.74-4.16	2.28	0.56	0.42-0.73
Negative	LSIL	1.05	0.07	1.56	1-year return^c^	1.71	1.63	1.02-2.60	0.19	2.71	0.68-10.79	2.16	1.39	0.82-2.33
Negative	ASC-US	0.04	0.01	0.24	3-year return^d^	0.02	0.51	0.13-2.02	<0.001	NA	NA	0.66	2.79	1.68-4.64
Negative	NILM	<0.001	<0.001	0.07	5-year return^e^	<0.001	NA	NA	<0.001	NA	NA	0.28	4.01	2.85-5.63
Overall		0.46	NA	NA	NA	1.42	3.09	2.92-3.26	0.53	NA	NA	1.88	NA	NA

Risks in KPNC cohort are from Didem Egemen et al.^17^ Risks determining the recommended management were bolded.^5,18^ The O/E values that are significantly deviates from 1.0 are in red.

^a^Treatment/colp. either treatment or colposcopy/biopsy is acceptable, the clinical action threshold is immediate CIN3+ risk at 25%-59%.

^b^Colp., colposcopy/biopsy, the clinical action threshold is immediate CIN3+ risk at 4%-24%. For women with HPV-negative/ASC-H and or AGC, colposcopy is recommended, because they are rare high-grade findings and their cancer risks are disproportionately high compared with CIN 3+ risk. When immediate CIN3+ risk is less than 4%, the surveillance interval is referred to 3-year CIN3+ or 5-year CIN3+ risk.

^c^1-year return, the clinical action threshold is 3-year CIN3+ risk ≥ 0.39% or 5-year CIN3+ risk ≥ 0.55%.

^d^3-year return, the clinical action threshold is 3-year CIN3+ risk less than 0.39% or 5-year CIN3+ risk at 0.15-0.54%.

^e^5-year return, the clinical action threshold is 5-year CIN3+ risk less than 0.15%. If 5-year CIN3+ risk is unavailable, management refers to 3-year CIN3+ risk threshold. *n* indicates number of females; O/E, observed/expected; CI, confidence interval; Immed., immediate; CIN, cervical intraepithelial neoplasia; NA, not applicable; KPNC, the Kaiser Permanente Northern California; WHUH, Wuhan Union Hospital.

Despite the differences observed between cohorts in terms of immediate risks, the action threshold at 4% of immediate CIN3+ risk showed a perfect portability, with all the subpopulations over the threshold being identical between cohorts (Table 5). Among them, hrHPV-positive/HSIL+ was associated with an immediate CIN3+ risk of 70.19% in our cohort, hitting the 60%-100% expedited treatment threshold, whereas it was 48.86% in the KPNC, which was within the threshold for treatment or colposcopy. Another exception to management inconsistency was women with hrHPV/AGC, who showed an immediate CIN3+ risk of 23.31% in our cohort, which is just under the 24% threshold for treatment or colposcopy. However, considering the high cancer risk of 17.23% in women with hrHPV/AGC, treatment or colposcopy should be recommended.

Also, we evaluated the portability of the 3-year CIN3+ risk, which serves as a reference for determining the surveillance interval in women with an immediate CIN3+ risk lower than 4%. Despite the insufficient follow-ups, the 3-year CIN3+ risk estimates remained robust in the entire cohort vs those with a follow-up beyond 3 years. Among the 12 subpopulations with varied screening test results, the O/E values range from 0.75 to 1.73, while all the 95% CIs include 1 (Table S2). Insufficient portability was observed in women who were hrHPV-positive/NILM (O/E, 0.56; 95% CI, 0.42-0.73), hrHPV-negative/ASC-US (O/E, 2.79; 95% CI, 1.68-4.64), and hrHPV-negative/NILM (O/E, 4.01; 95% CI, 2.85-5.63). With respect to management, more intensive surveillance with shorter intervals seems to be reasonable in the WHUH, especially for women with hrHPV-negative/ASC-US or who demonstrate completely negative screening results. Among these 2 subpopulations, the 3-year CIN3+ risk was 0.66% and 0.28% in the WHUH, respectively, whereas it was 0.24% and 0.07% in the KPNC, respectively (Table 5). When the ASCCP cutoff value for 1-year or 3-year return (0.39%, 3-year CIN3+ risk) was used, the hrHPV-negative/ASC-US women would be suggested to return at 1 year, and those women without abnormalities at 3 years, while their counterparts in the KPNC would both be suggested to have 3-year follow-up (<0.39%, 3-year CIN3+ risk) or 3-year and 5-year follow-up, respectively (according to the 5-year CIN3+ risk).

## Discussion

Cervical cancer is largely preventable through screening and vaccination. However, the current screening coverage falls significantly short of the goal of 70% set by WHO,^19^ and the disease burden remains uncontrolled globally. Opportunistic screening contributes to expanding screening coverage and is an integral part of cervical cancer prevention programs in less developed areas where the disease burden is high.^20,21^ Participants undergoing opportunistic screening often differ demographically from those undergoing organized screening. Nevertheless, they are often managed under the same guidelines due to a lack of specific data focusing on opportunistic settings. Here, we report the CIN3+ risks in an outpatient screening cohort of 96 318 Chinese females, as well as the projected outcomes of risk-based management and the portability of the ASCCP guidelines, constructing a detailed risk profile of opportunistic screening population, which may enhance informed decision-making and optimize screening performance against the backdrop of the cervical cancer elimination movement launched by WHO.

The cohort we studied was characterized by a higher median age of 42 years and a lower follow-up rate of 23%. Given unknown history before initial tests and unknown exact onset times of incident cervical lesions, we employed the prevalence-incidence mixture models to estimate risks. These models offer greater flexibility and precision compared to traditional methods, such as Kaplan–Meier and Turnbull analysis, by minimizing the underestimation of early stage risks and the overestimation of late-stage risks.^10,16,22^ The same statistical models were utilized in the KPNC study supporting the 2019 ASCCP guidelines, enabling direct comparisons between cohorts. In the WHUH cohort, the CIN3+ immediate risk was 1.42%, approximately 3 times higher than that in the KPNC cohort (0.46%). This discrepancy may largely stem from the higher prevalence of hrHPV in the WHUH cohort (17% vs 8% at the initial screening). A retrospective study reported an HPV prevalence of 15% among 14 492 outpatient women who visited a hospital in Wuhan.^23^ Additionally, outpatients are more likely to have conditions that compromise systemic and local immunity, increasing their risk of HPV persistence, CIN, or cervical cancer. Such conditions include HIV infection, immunosuppressive medications for inflammatory bowel disease, and an altered vaginal microbiome.^24^ Furthermore, the potential influence of racial factors cannot be excluded.^25^ WHUH is a medical center in central China, located in Wuhan, the capital of Hubei Province. Wuhan is a major city with a population of over 13 million, where organized screening programs have been implemented since 2009.^26,27^ Similar hrHPV prevalence rates have been observed among outpatient women in other large Chinese cities, such as Beijing (22.7%)^28^ and Shanghai (17.9%),^29^ which seem to be elevated over the general Chinese population. A meta-analysis enrolling 853,945 Chinese women with normal cervix revealed a hrHPV prevalence of 11.3%.^30^ However, we failed to identify any data on CIN risks based on the prevalence-incidence mixture models in other Chinese populations, and comparisons between domestic cohorts are difficult at this stage.

In the present study, we chose CIN3+ as the primary endpoint because (1) the primary goal of cervical cancer screening is to detect and treat cervical precancer to prevent cancer, rather than to detect invasive cancer, (2) the pathologic diagnosis of CIN3 is more robust than that of CIN2, (3) CIN3 is widely advised to be treated immediately, as it associated with an up to 30% risk of progression to cervical cancer,^31^ and (4) it is also the primary endpoint in the KPNC study. Given the epidemiological variations across countries, threshold selection and primary endpoints should be adapted to local contexts. Consequently, our study included risk estimations for both CIN2 and invasive cancer and projected outcomes from different colposcopy/biopsy referral thresholds as shown in Table 4. In high-incidence, resource-limited settings (eg, Sub-Saharan Africa and parts of Asia), to avoid overloading health care system, employing a higher threshold of CIN3+ for further intervention or using invasive cancer as primary endpoint for risk stratification may be more appropriate. We see a CIN3+ immediate risk of 4% as a reasonable threshold for Chinese populations because of both high efficiency (5 colposcopies for 1 CIN3+ detected) and low CIN3+ misdiagnose rate (180/10 000) (Table 4), and the demands for colposcopy examinations can be sufficiently backed by our medical infrastructure. In addition, certain subpopulations can vary in risk of being over- or undertreated. For example, younger populations are more likely to be affected by overtreatment, due to high HPV prevalence and low CIN3+ risk, while HPV-negative populations are more likely to be undertreated, especially in HPV-primary screening. Therefore, further risk estimates in such subpopulations are necessary to optimize their management.

HPV infection status is crucial for stratifying CIN risks and guiding treatment. In our study, the CIN3+ immediate risk was 14.14% among hrHPV-positive women, significantly higher than the 0.12% observed in hrHPV-negative women. These findings support the adoption of primary HPV testing in opportunistic screening, particularly in resource-limited settings, as it allows for the omission of initial cytology in HPV-negative women compared to co-testing. Moreover, we found that HPV genotyping provides additional value in estimating risks and tailoring management for HPV-positive women. We found that the declines in CIN3+ and cancer risks from HPV18 to HPV16, then to other hrHPV were sharp and treatment relevant. Specifically, the management of women with hrHPV-positive ASC-US can be downgraded from direct colposcopy referral for all hrHPV-positive to 1-year return for other hrHPV-positive. Similar changes in recommendations were also suggested by a study involving 7463 hrHPV-positive women in the KPNC cohort who had undergone partial HPV genotyping tests (Cobas and Onclarity).^32^ Given different hrHPV types vary in prevalence, evolutionary fitness, and risks of causing precancers and cancers,^33-35^ more detailed genotyping should be incorporated in screening to further fine-tune the stratification of hrHPV-positive women.

Despite significant discrepancies in risk levels between the cohorts of WHUH and KPNC, the corresponding risk-based management recommendations were largely consistent. When stratified by HPV and cytology results, some of the CIN3+ immediate risks in our cohort were higher than those of KPNC, but most fell within the same bounds of the recommended management. A similar pattern was observed in the never/rarely/unknown population of the CDC PNBCCEDP and the population of the ATHENA study.^7^ However, there are exceptions. For women with hrHPV-positive/HSIL+ results in our cohort, expedited treatment may be preferred, unlike the treatment/colposcopy approach in the KPNC cohort, due to the significantly higher immediate CIN3+ risk of 70%, exceeding the 60% threshold for treatment. Additionally, the immediate cancer risk of 30% and the elevated risk of not returning for colposcopy among outpatients further support this approach. Among women in the CDC PNBCCEDP who had been poorly screened, hrHPV-positive/HSIL+ results were also associated with an immediate CIN3+ risk greater than 60%.^36^ A recent retrospective multicohort study in China (including 10 055 records across 9 cohorts) showed 87.1% sensitivity and 82.5% specificity for CIN3+ detection using the ASCCP app’s threshold,^37^ which is in line with the high portability of the 4% threshold revealed by the prevalence-incidence mixture model in our study, suggesting a broader applicability of the threshold in China.

Moreover, it is important to note the limited portability of the KPNC 3-year CIN3+ risk and follow-up intervals for women with hrHPV-negative/ASC-US or negative co-tests. In these 2 subgroups, the 3-year CIN3+ risk was significantly higher in WHUH compared to KPNC, with O/Es of 2.79 and 4.01, respectively. This implies a follow-up interval of 1 year (for a 3-year CIN3+ risk <0.39%) and 3 years (for a 3-year CIN3+ risk ≥ 0.39%), both of which are shorter than those recommended in the KPNC.^18^ In contrast to the CIN3+ risks, cancer risks increased disproportionately in the WHUH cohort. The 3-year cancer risk for women with hrHPV-negative/ASC-US or negative co-tests was 0.32% and 0.06%, respectively (data not shown), approximately 10 times higher than in the KPNC cohort. Another concern is that the risks might be underestimated, as populations lost to follow-up often have higher risks.^38^ Besides, the opportunistic screening approach is inherently associated with a higher risk of loss to follow-up with prolonged intervals, which should also be considered when determining follow-up intervals.^39^ Collectively, we would recommend 1-year return for outpatients with sole ASC-US or normal co-tests in regions where resources are sufficient. However, in settings with constrained resources, frequent surveillance in low-risk or borderline cases may present technical and economic challenges and overburden both the healthcare system and patients.

To the best of our knowledge, the WHUH cohort is one of the largest opportunistic screening cohorts, with high HPV prevalence and minimal vaccination coverage, and this is the first study to estimate the risks associated with opportunistic screening cohorts using prevalence-incidence mixture models and to validate risk-based management in regions outside the United States.^40^ HPV vaccination was only nationally implemented during 2016-2018, with Hubei Province achieving merely 1.44% coverage among women aged 9-45 by 2020. This epidemiological context underscores the continued importance of prevaccination population studies for guiding screening strategies. A recent nationwide 9-cohort study evaluated ASCCP performance utilizing the ASCCP mobile application, which generates individualized risk estimates based on KPNC cohort data in response to input screening results and histories.^37^ In contrast, we employed the prevalence-incidence mixture model to derive population-level risk estimates specific to the WHUH cohort while maintaining broader screening policy relevance.

However, this study has several limitations. First, the data were collected retrospectively from a single institution with potential selection bias. In an opportunistic hospital-based setting like WHUH, women may seek screening due to symptoms or underlying concerns, which could inflate baseline CIN3+ risk estimates. Second, our study is unable to address the thresholds in other populations such as younger populations or immunosuppressed individuals or those under HPV-primary screening. Thus, the results should not be overinterpreted or generalized to other populations without external verification. Third, the 3-year and 5-year risks might be underestimated because of insufficient follow-up data in the cohort studied. Fourth, for the same reason, the risk estimations were limited to women with unknown histories, and the influence of screening history on risk estimations and risk-based management was not evaluated.

In conclusion, the WHUH cohort, which is representative of opportunistic screening populations in China’s large cities, had higher risks of cervical precancerous lesions, which can be stratified by the presence or absence of hrHPV infection and the subtypes of infected virus for informing risk-based management. Despite the higher risks in our cohort, the ASCCP clinical action threshold (a CIN3+ immediate risk >4%) remains portable. But for women with minimal abnormalities or normal results, shortened follow-up intervals should be considered if sufficient resources are available.

## Supplementary Material

oyaf197_suppl_Supplementary_Figures_1

oyaf197_suppl_Supplementary_Tables_1

oyaf197_suppl_Supplementary_Tables_2

## Data Availability

The data underlying this article will be shared on reasonable request to the corresponding author.
