# Supplementary material for: Risk assessment in a Chinese cohort of 96 318 females undergoing opportunistic cervical cancer screening
Source: Oncologist. 2025 Jul 14;30(7):oyaf197. doi: 10.1093/oncolo/oyaf197 (PMC12259530; doi:10.1093/oncolo/oyaf197)
Supplement: oyaf197_suppl_Supplementary_Figures_1 [file oyaf197_suppl_supplementary_figures_1.docx]

**
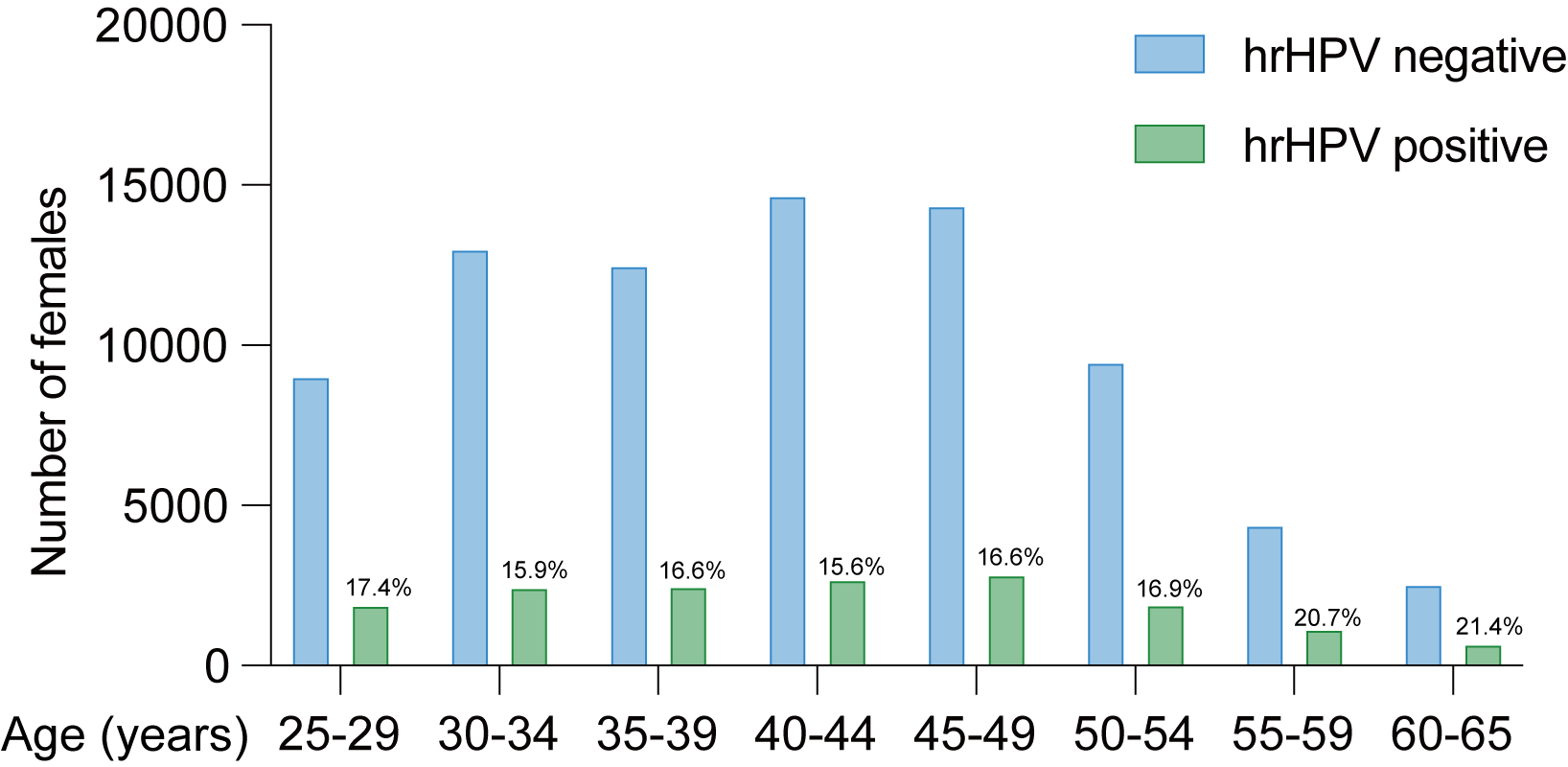
**

**Supplementary Figure 1.** The age distribution and hrHPV prevalence in women at their first visit for cervical cancer screening at WHUH.
