# Supplementary material for: Risk assessment in a Chinese cohort of 96 318 females undergoing opportunistic cervical cancer screening
Source: Oncologist. 2025 Jul 14;30(7):oyaf197. doi: 10.1093/oncolo/oyaf197 (PMC12259530; doi:10.1093/oncolo/oyaf197)
Supplement: oyaf197_suppl_Supplementary_Tables_1 [file oyaf197_suppl_supplementary_tables_1.docx]

**Supplementary Table 1.** Comparisons of basic characteristics between the KPNC and WHUH cohorts

| **Characteristics** | **KPNC** | **WHUH** |
| --- | --- | --- |
| Screening participants |  |  |
| Study period | 2003-2017 | 2011-2020 |
| Age at first screening, years | 25-65 | 25-65 |
| Number of women included*^a^* | 1,546,462 | 96,318 |
| Screening more than once, *n* (%) | 920,066 (59·49) | 22,125 (22·97) |
| Vaccination against HPV infection | rare | rare |
| Screening method |  |  |
| Screening strategy | HPV and cytology cotesting | HPV and cytology cotesting |
| Cytology | BD SurePath | BD SurePath |
| HPV test | Hybrid Capture 2 or Onclarity | Luminex 200 |
| HPV genotyping | partial HPV genotyping (16,18) or none | 16, 18, 31, 33, 35, 39, 45, 51, 52, 56, 58, 59, 68, 26, 66, 53, 82 |
| Screening results, *n* (%)*^a^* |  |  |
| hrHPV positive | 126,429 (8·18) | 16,215 (16·83) |
| HSIL+ | 3980 (0·26) | 1163 (1·21) |
| AGC | 977 (0·06) | 416 (0·43) |
| ASC-H | 3766 (0·24) | 427 (0·44) |
| LSIL | 23,659 (1·53) | 2252 (2·34) |
| ASC-US | 30,506 (1·97) | 2975 (3·09) |
| NILM | 63,541 (4·11) | 8982 (9·33) |
| hrHPV negative | 1420033 (91·82) | 80103 (83·17) |
| HSIL+ | 183 (0·01) | 137 (0·14) |
| AGC | 2275 (0·15) | 1277 (1·33) |
| ASC-H | 791 (0·05) | 661 (0·69) |
| LSIL | 3300 (0·21) | 1343 (1·39) |
| ASC-US | 25,331 (1·64) | 10,084 (10·47) |
| NILM | 1,388,153 (89·76) | 66,601 (69·15) |
| Abnormal cytology (≥ASC-US) | 94,768 (6·13) | 20,735 (21·53) |
| Cervical lesions, *n* (%)*^a^* |  |  |
| CIN2+ | 21,395 (1·38) | 2270 (2·36) |
| CIN3+ | 8473 (0·55) | 1321 (1·37) |
| Cancer | 591 (0·04) | 486 (0·50) |

Data of KPNC cohort are obtained from Didem Egemen et al.^1^

*^a^*females with unknown prior HPV test results. KPNC, Kaiser Permanente of Northern California; WHUH, Wuhan Union Hospital; CIN, cervical intraepithelial neoplasia; hrHPV, high-risk human papillomavirus.
