# Supplementary material for: Risk assessment in a Chinese cohort of 96 318 females undergoing opportunistic cervical cancer screening
Source: Oncologist. 2025 Jul 14;30(7):oyaf197. doi: 10.1093/oncolo/oyaf197 (PMC12259530; doi:10.1093/oncolo/oyaf197)
Supplement: oyaf197_suppl_Supplementary_Tables_2 [file oyaf197_suppl_supplementary_tables_2.docx]

**Supplementary Table 2** Sensitive analysis: The 3-year CIN3+ risks in all WHUH screening females (*n*=96,318) and females with follow-up beyond 3 years (*n*=8861).

| **hrHPV** | **Cytology** | **All women** | | |  | | | **Women with follow-up beyond 3 years** | | | | |
| --- | --- | --- | --- | --- | --- | --- | --- | --- | --- | --- | --- | --- |
|  |  | ***n*** | **%** | **Risks** |  | | ***n*** | | **%** | **Risks** | **O/E** | **95% CI** |
| Positive | HSIL+ | 1163 | 1·21 | 70·45 |  | | 148 | | 1·67 | 71·72 | 1·02 | 0·91-1·12 |
|  | AGC | 416 | 0·43 | 23·31 |  | | 40 | | 0·45 | 30·77 | 1·32 | 0·81-2·05 |
|  | ASC-H | 427 | 0·44 | 29·79 |  | | 51 | | 0·58 | 22·27 | 0·75 | 0·44-1·22 |
|  | LSIL | 2252 | 2·34 | 10·57 |  | | 307 | | 3·46 | 11·34 | 1·07 | 0·78-1·47 |
|  | ASC-US | 2975 | 3·09 | 4·95 |  | | 325 | | 3·67 | 5·58 | 1·13 | 0·71-1·77 |
|  | NILM | 8982 | 9·33 | 2·28 |  | | 892 | | 10·07 | 2·54 | 1·11 | 0·73-1·69 |
| Negative | HSIL+ | 137 | 0·14 | 47·66 |  | | 15 | | 0·17 | 40·00 | 0·84 | 0·43-1·43 |
|  | AGC | 1277 | 1·33 | 2·81 |  | | 77 | | 0·87 | 3·70 | 1·32 | 0·43-3·96 |
|  | ASC-H | 661 | 0·69 | 3·04 |  | | 86 | | 0·97 | 2·33 | 0·77 | 0·19-2·95 |
|  | LSIL | 1343 | 1·39 | 2·16 |  | | 169 | | 1·91 | 3·64 | 1·68 | 0·76-3·66 |
|  | ASC-US | 10,084 | 10·47 | 0·66 |  | | 1121 | | 12·65 | 1·14 | 1·73 | 0·99-3·01 |
|  | NILM | 66,601 | 69·15 | 0·28 | |  | 5630 | | 63·54 | 0·30 | 1·07 | 0·64-1·78 |
